# Identification of Key Biomarkers Associated with Immunogenic Cell Death and Their Regulatory Mechanisms in Severe Acute Pancreatitis Based on WGCNA and Machine Learning

**DOI:** 10.3390/ijms24033033

**Published:** 2023-02-03

**Authors:** Zhengjian Wang, Jin Liu, Yuting Wang, Hui Guo, Fan Li, Yinan Cao, Liang Zhao, Hailong Chen

**Affiliations:** 1 Department of General Surgery, The First Affiliated Hospital of Dalian Medical University, Dalian 116000, China; 2 Institute (College) of Integrative Medicine, Dalian Medical University, Dalian 116000, China; 3 Laboratory of Integrative Medicine, The First Affiliated Hospital of Dalian Medical University, Dalian 116000, China

**Keywords:** severe acute pancreatitis, immunogenic cell death, WGCNA, LASSO, machine learning, immune cell infiltration, short-chain fatty acids

## Abstract

Immunogenic cell death (ICD) is a form of programmed cell death with a strong sense of inflammatory detection, whose powerful situational awareness can cause the reactivation of aberrant immunity. However, the role of ICD in the pathogenesis of severe acute pancreatitis (SAP) has yet to be investigated. This study aims to explore the pivotal genes associated with ICD in SAP and how they relate to immune infiltration and short-chain fatty acids (SCFAs), in order to provide a theoretical foundation for further, in-depth mechanistic studies. We downloaded GSE194331 datasets from the Gene Expression Omnibus (GEO). The use of differentially expressed gene (DEG) analysis; weighted gene co-expression network analysis (WGCNA) and least absolute shrinkage and selection operator (LASSO) regression analysis allowed us to identify a total of three ICD-related hub genes (*LY96*, *BCL2*, *IFNGR1*) in SAP. Furthermore, single sample gene set enrichment analysis (ssGSEA) demonstrated that hub genes are closely associated with the infiltration of specific immune cells, the activation of immune pathways and the metabolism of SCFAs (especially butyrate). These findings were validated through the analysis of gene expression patterns in both clinical patients and rat animal models of SAP. In conclusion, the first concept of ICD in the pathogenesis of SAP was proposed in our study. This has important implications for future investigations into the pro-inflammatory immune mechanisms mediated by damage-associated molecular patterns (DAMPs) in the late stages of SAP.

## 1. Introduction

Acute pancreatitis (AP) is an injurious inflammatory disease caused by the abnormal function of exocrine pancreas, primarily due to biliary calculi, overeating, metabolic and immune dysfunction, etc., which is one of the common acute and critical conditions in general surgery [1,2]. Without timely diagnosis and treatment, about 20–35% of AP patients will progress to severe acute pancreatitis (SAP). SAP is characterized by persistent multi-organ failure and microcirculatory disturbances (particularly in the intestines, lungs, and kidneys) and has a mortality rate of up to 30% [3,4,5,6]. Given the insidious onset of SAP, its numerous complications, and its high mortality rate, it is essential to actively investigate the molecular mechanisms that trigger the expansion of the systemic inflammatory response during the progression of SAP, especially the immune factors, for subsequent in-depth basic research and improvement of the prognosis of SAP patients [7,8].

SAP is often associated with intestinal barrier dysfunction and sustained activation of pro-inflammatory immunity, while the specific regulatory mechanics still need to be completed. The composition and characteristics of the gut microbiota in SAP have been demonstrated to be significantly altered by an increase in the stereotypic resistance of parthenogenic pathogens such as *Enterococcus*, *Enterobacter*, *Streptococcus*, and *Shigella*, which is also considered to be a “trigger” for changes to the intestinal immune barrier and the body’s immune defense in SAP [9,10,11,12]. In addition, the gut microbiota and its derived small molecule metabolites also play an equally pivotal role in the host’s normal defense and immune response [13,14]. Short-chain fatty acids (SCFAs) have been shown to be produced by the fermentation of dietary fiber and polysaccharides by the dominant intestinal genera (*Bacteroidetes*, *Roseburia*, *Parabacteroides*, *Akkermansia*, *Prevotella*). These small molecules have various functions such as: promoting renewal and repair of damaged intestinal epithelial cells; inducing polarisation of M2-type macrophages; increasing the number of Tregs cells; promoting energy conversion; alleviating damage-associated molecular patterns (DAMPs); and protecting against multi-organ functional damage in SAP [15,16,17,18]. Several studies have proven that the concentration of SCFAs is significantly lower in SAP and is clearly correlated with SAP-associated multi-organ failure [15,19,20].

Immunogenic cell death (ICD), defined as a specific variant of regulatory cell death (RCD), drives adaptive immune responses and establishes long-term immune memory in the host. ICD has two main properties: (i) antigenicity, defined as having antigenic determinants not yet covered by central immune tolerance, is capable of inducing initial T-cell clones that activate cytotoxic T-lymphocyte (CTL)-driven adaptive immunity; (ii) adjuvanticity, which facilitate the recruitment and activation of antigen presenting cells following cell death [21,22,23,24]. Interestingly, the lack of antigenic determinants in the early stage of AP activates inflammation but not an adaptive immune response. Given the massive release of inflammatory mediators, dying cells exhibit sufficient antigenicity such that their death leads to an adaptive immune response, orchestrated by CTL, which triggers immune memory, culminating in a persistent cytokine storm and immune infiltration. Stressors that can cause ICD in SAP include pathogenic bacteria and their pathogen-associated molecular patterns (PAMPs); abnormal epigenetic modifications due to reduced SCFAs (the abnormal expression of histone deacetylases; DNA methyltransferases, etc.); inadequate energy supply; inflammation; toxins and accumulation of reactive oxygen species (ROS) [21,25]. ICD occurs in conjunction with the production of corresponding signaling molecules, damage-associated molecular patterns (DAMPs), and cause an overload of immune regulation against dead cells [26,27,28]. The most representative ones are calreticulin, high mobility group box protein 1, ATP molecules, type I IFN, heat shock proteins, etc. [29,30]. These DAMPs are recognized by pattern recognition receptors (PRRs) on the cell surface and function as modulators of adaptive immune responses [31,32,33,34]. Given the immune surveillance and regulatory functions of ICDs, they are beneficial in the immunotherapy of many tumors. However, the side-effects of the consequential immune hyperactivity that result from having sufficient prerequisites to trigger ICD during SAP should also be considered [35,36,37,38,39].

With the continuous development of high-throughput transcriptomic technologies, such as microarray and sequencing, interrogating the vast amount of data available for the molecular targets that play an important role in the microregulatory mechanisms of disease progression is certainly a challenge for researchers. Weighted gene co-expression network analysis (WGCNA), an unbiased systems biology analysis method, aims to find co-expressed gene modules and explore the associations between gene networks and phenotypes of interest, as well as identifying core genes in the network [40,41,42]. The least absolute shrinkage and selection operator (LASSO) is a data-mining method commonly used in multiple-linear regression to streamline models by adding penalty functions and continuously compressing coefficients to avoid collinearity and overfitting [43]. Therefore, WGCNA combined with LASSO regression can effectively screen for the most valuable biomarkers associated with ICD during SAP.

To the best of our knowledge, the potential roles of ICD in the pathogenesis of SAP have not been reported. This paper aims to explore the key biomarkers associated with ICD in SAP and how they relate to immune cell infiltration, the activation of immune pathways, and the metabolism of SCFAs. Firstly, we extracted microarray datasets and the corresponding clinical information from the Gene Expression Omnibus (GEO) database of SAP patients and healthy controls for differentially expressed gene (DEG) analysis, and then used WGCNA to screen for the co-expressed gene modules most associated with the SAP phenotype. Secondly, we applied LASSO regression to further refine the intersecting genes of the screened module genes and ICD-related genes to obtain the three most valuable hub genes and evaluated their efficacy in terms of diagnostic value from an ICD perspective, which fully illustrates the importance of ICD in the development of SAP. This allowed us to be the first to quantify the proportion of immune cells in samples from SAP patients and healthy controls using ssGSEA (single-sample gene set enrichment analysis) and investigated the association of the identified biomarkers with the metabolism-related pathways of SCFAs. Finally, we utilized quantitative real-time PCR (qRT-PCR) and immunohistochemistry (IHC) to validate the expression of hub genes in SAP patients and rat models, providing a fresh perspective on the role of ICD in the pathogenesis of SAP and contributing to subsequent basic intervention studies.

## 2. Results

### 2.1. DEGs Screening

**Figure 1:**
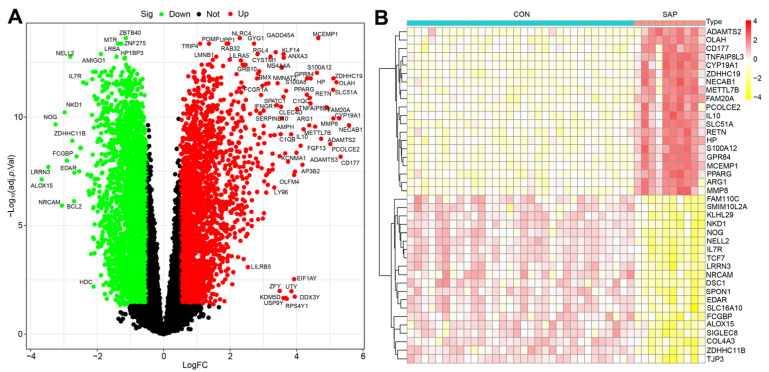
Volcano plot showing DEGs in SAP patients versus healthy controls (**A**) and the heatmap of TOP20 differential genes (upregulated and downregulated, (**B**)).

A total of 5601 DEGs were identified in this study using the GSE194331 dataset (containing sample data from 10 SAP patients and 32 healthy individuals) based on the criteria of |logFC| > 0.5 and adjusted *p* < 0.05, including 2541 upregulated genes and 3060 downregulated genes (Figure 1A). The top 20 genes from the upregulated and downregulated genes were selected separately for the heat map, with red indicating upregulated genes and yellow indicating downregulated genes (Figure 1B).

### 2.2. Construction of Co-Expression Network and Identification of SAP Core Genes

**Figure 2:**
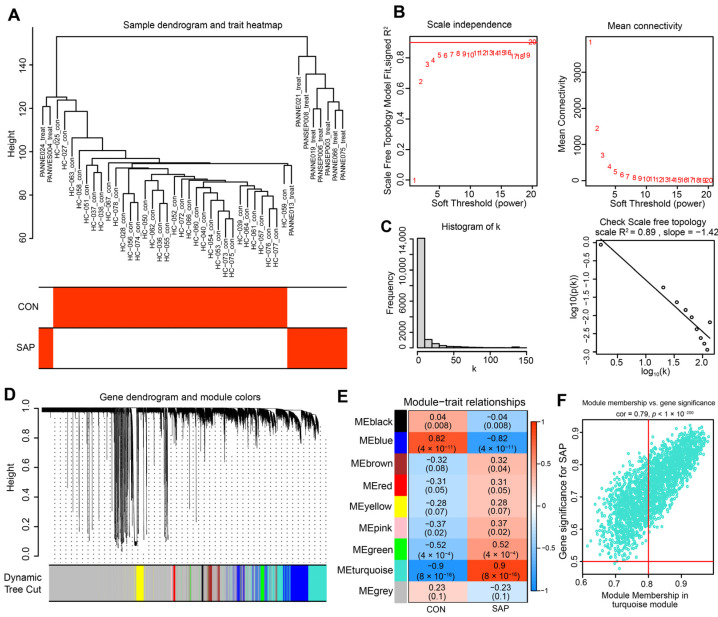
Construction of the co-expression network. (**A**) The sample dendrogram and feature heat map were drawn based on the Euclidean distance using the average clustering method for hierarchical clustering of samples, with each branch representing a sample, Height in the vertical coordinate being the clustering distance, and the horizontal coordinate being the clinical grouping information. (**B**) Soft threshold (power = 20) and scale-free topology fit index (R^2^ = 0.89). (**C**) Histogram of connectivity distribution. The scale-free topology is checked at a soft threshold of 20. (**D**) Gene hierarchy tree-clustering diagram. The graph indicates different genes horizontally and the uncorrelatedness between genes vertically, the lower the branch, the less uncorrelated the genes within the branch, i.e., the stronger the correlation. (**E**) Heatmap showing the relations between the module and SAP features. The values in the small cells of the graph represent the two-calculated correlation values cor coefficients between the eigenvalues of each trait and each module as well as the corresponding statistically significant *p*-values. Color corresponds to the size of the correlation; the darker the red, the more positive the correlation; the darker the green, the more negative the correlation. (**F**) Scatter plot between gene salience (GS) and module members (MM) in turquoise.

To further precisely excavate the central genes associated with the SAP phenotype, we constructed a gene co-expression network using the WGCNA algorithm. The sample hierarchical cluster analysis results showed good clustering among the samples, with no significant outliers (Figure 2A). The soft threshold was set to 20 to satisfy the scale-free topology of the network, where the corresponding R^2^ was 0.89 and the average connectivity was high (Figure 2B,C). A gene hierarchy clustering dendrogram was constructed by gene correlation, and a total of nine similar gene modules were identified (Figure 2D). Subsequently, we finally identified the “turquoise” module, containing 1548 genes, as the most clinically valuable module in SAP (Cor = 0.9, *p* = 8 × 10^−16^) based on the correlation of module feature values with SAP phenotypes (Figure 2E). The scatter plot (Figure 2F) shows a strong correlation between GS and MM in the “turquoise” module (Cor = 0.79, *p* = 1 × 10^−200^).

### 2.3. Selection and Functional Enrichment Analysis of ICD-Related Genes in SAP

**Figure 3:**
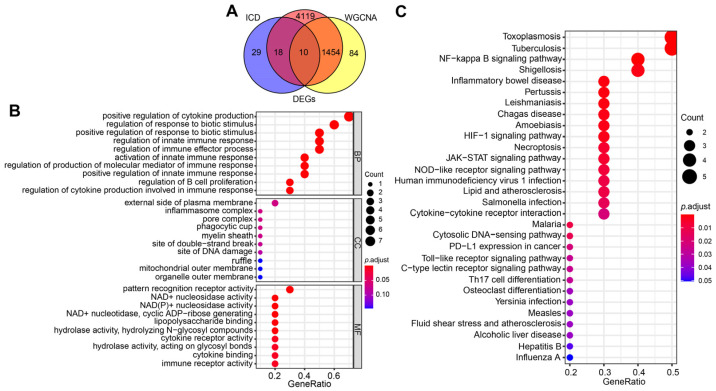
Screening for ICD-related signature genes in SAP and functional enrichment analysis. (**A**) Venn diagram of the intersection of DEGs, turquoise module genes, and ICD-related genes. (**B**) GO functional annotation of signature genes. (**C**) Functional annotation of the Kegg signaling pathway of signature genes. For all enriched GO and KEGG terms, *p* < 0.05.

To explore the regulatory role of ICD in the pathogenesis of SAP, we first intersected DEGs, turquoise module genes, and ICD-related genes to obtain a total of 10 signature genes (Figure 3A). Subsequently, we performed functional enrichment analysis of these signature genes to explore the biological functions and potential pathways associated with ICD that occur in SAP. The results of GO enrichment analysis showed that the signature genes mainly affect host inflammation (positive regulation of cytokines, production of inflammatory complexes), immune regulation (activation of the innate immune response, B-cell infiltration, enhanced Tregs cell function), and PRRs activity (Figure 3B). The results of the KEGG signaling pathway analysis demonstrated that the signature genes were most enriched in inflammatory pathways (e.g., NF-κB, JAK-STAT, NOD-like receptors, cytokine receptors, etc.); immune-related pathways (infiltration of Th17 cells, intercellular communication); microbial infection and intestinal homeostasis (HIF-1 signaling pathway) (Figure 3C). These are in line with the inflammatory “storm”, microbiota disruption, and DAMP production that cause SAP, and they indirectly point to the ICD as a potential major component in the pathogenesis of pro-inflammatory immunity during SAP.

### 2.4. Identification of the Hub Genes Most Associated with ICD Occurrence in SAP

**Figure 4:**
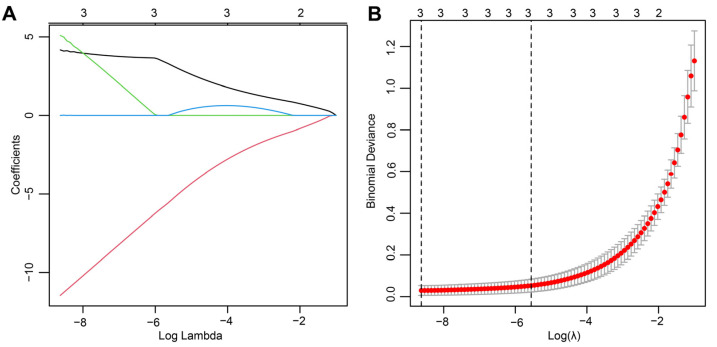
The final hub genes were identified by LASSO regression analysis. (**A**) Path diagram of the LASSO coefficients for the three hub genes in SAP. Each curve represents the trajectory of each hub gene, the vertical coordinate is the value of the gene, the lower horizontal coordinate is log(λ), and the upper horizontal coordinate is the number of non-zero hub genes in the model at this time. (**B**) LASSO regression cross-validation curve. Optimal λ values were determined using 10-fold cross-validation.

**Figure 5:**
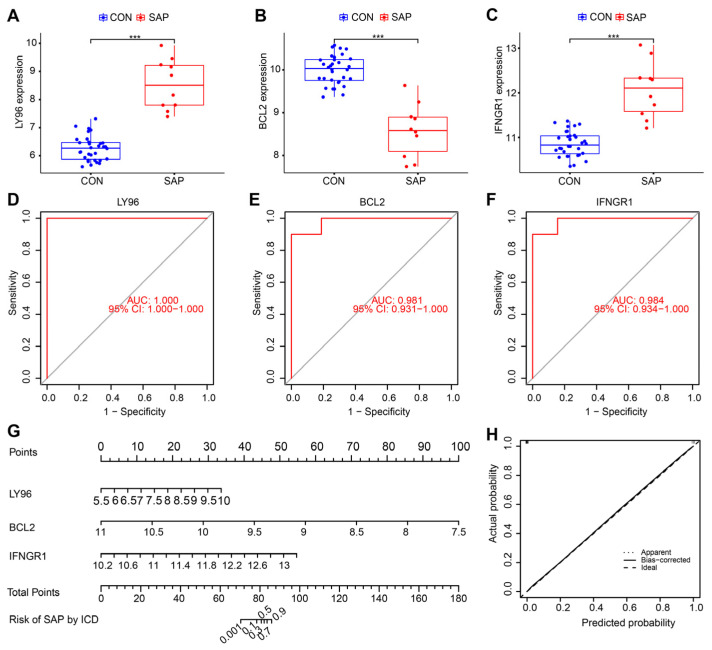
Validation of the importance of ICD in the pathogenesis of SAP through the hub gene. (**A**–**C**) Expression levels of three hub genes in SAP patients compared with healthy controls, *** *p* < 0.01. (**D**–**F**) ROC analysis of three hub genes. (**G**) Nomogram for measuring the significance of ICD in SAP based on hub genes. (**H**) Calibration curve plot for the nomogram. The X-axis represents the predictable probability, and the Y-axis represents the actual probability. Perfect prediction corresponds to the ideal dashed line. The apparent dashed line represents the entire queue, bias-corrected solid line is bias-corrected by bootstrapping (1000 repetitions) and represents the observed performance of the nomogram.

To further streamline the important characteristic variables to identify the core genes, we performed LASSO regression analysis based on lambda. min (0.0001793774), which identified three hub genes (*LY96*, *BCL2*, and *IFNGR1)* as being most representative of the genes associated with ICD occurrence in SAP (Figure 4A,B). Compared with healthy controls, *BCL2* expression was downregulated, whereas *LY96* and *IFNGR1* expression was upregulated in SAP patients (all *p* < 0.001) (Figure 5A–C). In the ROC curve analysis, the sensitivity and specificity of the three hub genes for assessing the importance of ICD in SAP by analyzing their AUC values, and the AUC values for all three genes were greater than 0.98, suggesting that these genes show superior representation for ICD (Figure 5D–F). Subsequently, to further assess the clinical applicability of the binomial regression model, we constructed a nomogram showing the hub genes (Figure 5G). The calibration plots of the nomogram model proved an excellent fit between the predictions of the column line graph and the actual probabilities, which provided strong evidence for the reliability of the presence of ICD in SAP (Figure 5H).

### 2.5. Immune Cell Infiltration/Functions and Its Association with Hub Genes

**Figure 6:**
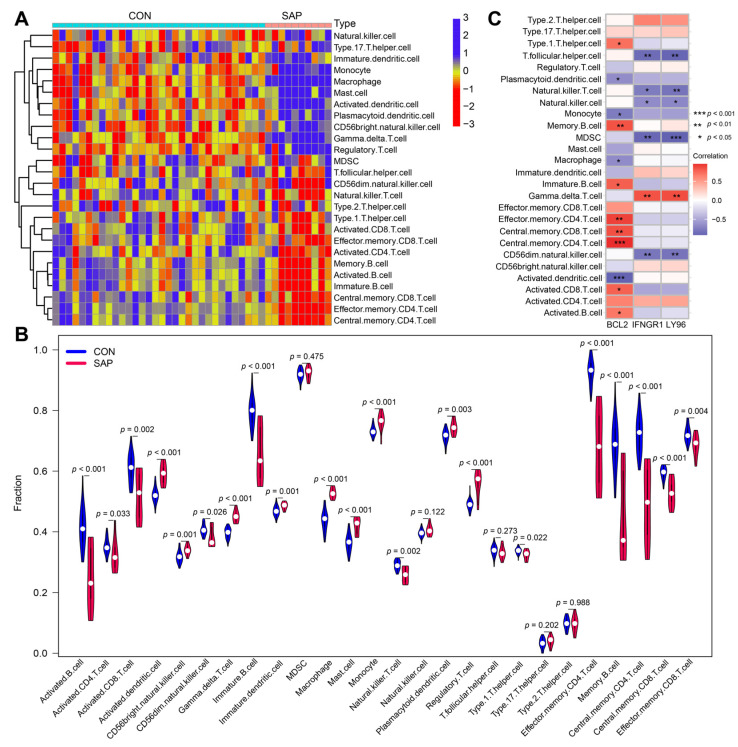
Analysis of immune infiltration in SAP patient samples and its correlation with hub genes using ssGSEA. (**A**) Heat map showing the immune scores of 26 immune cells. Red indicates immune cell infiltration and blue indicates suppressed immune cells. (**B**) Violin plot showing immune scores of 26 immune cells in SAP patients and healthy controls. (**C**) Correlation between immune cell infiltration and three hub genes. * *p* < 0.05, ** *p* < 0.01, *** *p* < 0.001.

**Figure 7:**
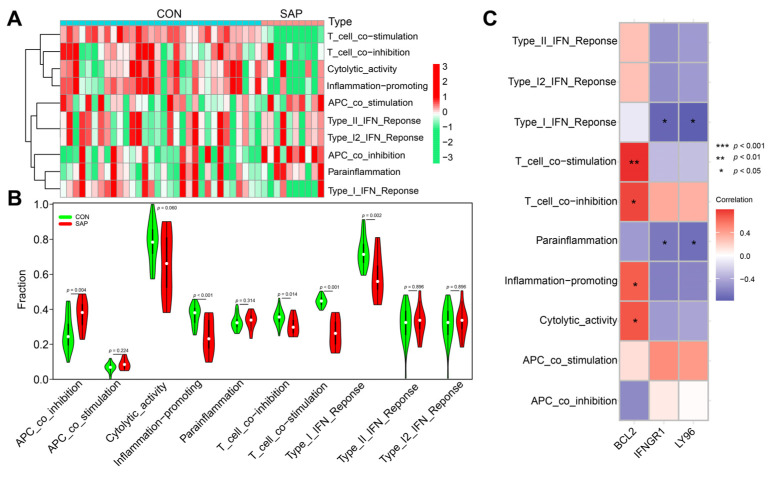
Analysis of immune functions in SAP patient samples and their correlation with hub genes using ssGSEA. (**A**) Heatmap showing immune scores for 10 immune functions in SAP patients and healthy controls. Red indicates activation of this immune function, green indicates functional inhibition. (**B**) Violin plot showing immune scores for 10 immune functions in SAP patients and healthy controls. (**C**) Association between immune cell function and three hub genes; * *p* < 0.05, ** *p* < 0.01.

Differences in the expression of immune infiltrate between SAP patients and healthy controls were further investigated using ssGSEA. Figure 6A and Figure 7A show the distribution of 26 immune cells and 10 immune functions in the GSE194331 dataset, respectively. In the heat map, the horizontal axis indicates the name of the sample and the vertical axis indicates the different immune cells or immune functions; the clustering tree on the left of the heat map indicates the clustering of the units on the vertical axis. Furthermore, the shades of color represent the degree of cellular infiltration or functional radicalization. The more concentrated the colors of the different samples in the same group, the more representative the data are. We observed a significantly higher infiltration of activated CD4 T cell, activated dendritic cell, CD56bright natural killer cell, gamma delta T cell, macrophage, mast cell, monocyte, and plasmacytoid dendritic cell, regulatory T cell, T follicular helper cell, type 17 T helper cells, and type 2 T helper cells in samples from SAP patients, suggesting a key role for these cells in the progression of SAP (Figure 6B). Correlation analysis showed that *BCL2* was positively correlated with central memory CD4 T cell, central memory CD8 T cell, effector memory CD4 T cell, memory B cell and type 1 T helper cells involved in the adaptive immune response and negatively correlated with activated dendritic cells (all *p* < 0.05) (Figure 6C). In contrast, *LY96* and *IFNGR1* were significantly positively correlated with gamma delta T cell and significantly negatively correlated with CD56dim natural killer cell and T folic helper cell (all *p* < 0.01). These results simultaneously provide further evidence for the cellular–molecular mechanics by which ICD plays a regulatory role in the progression of SAP. Furthermore, differential analysis of immune function showed that T cell co-stimulation, T cell co-inhibition, inflammation-promotion, type I IFN response, and cytolytic activity were closely associated with ICD in SAP (Figure 7B,C).

### 2.6. Correlation Analysis of Hub Genes and SCFAs

**Figure 8:**
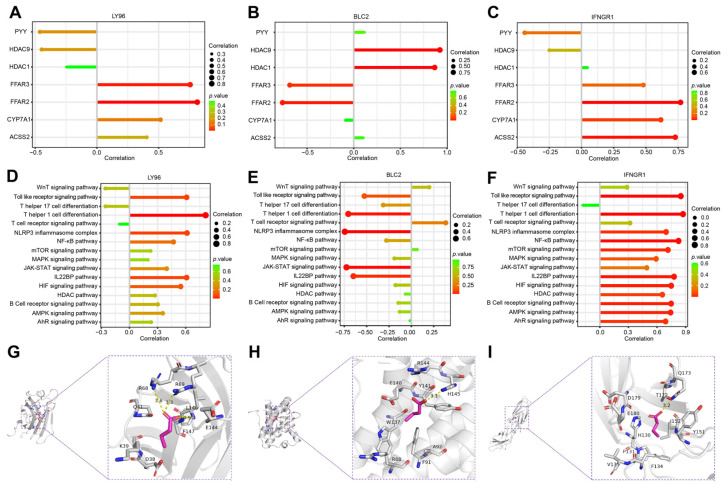
Correlation of hub genes with SCFAs in SAP patient samples analyzed using ssGSEA. (**A**–**C**) Lollipop plots of three hub genes with genes related to the metabolism of SCFAs. (**D**–**F**) Lollipop plots of three hub genes and the star pathway associated with the metabolism of SCFAs. (**G**–**I**) Molecular docking assemblies of three hub genes interacting with butyric acid. Hub genes (*LY96*, *BCL2*, *IFNGR1*) interact with butyric acid mainly through hydrogen bonding and hydrophobic forces, with binding energies of −3.9 kcal/mol, −3.5 kcal/mol, and −3.9 kcal/mol, respectively. The lower the binding energy, the stronger the stability.

Current studies have confirmed that SCFAs have important protective functions during SAP [15]. To investigate whether ICD is involved in the microregulation of SCFAs in SAP, we explored the association of hub genes with genes related to the metabolism of SCFAs in SAP based on the GSE194331 dataset. Correlation coefficient plots and lollipop plots showed that *LY96* and *IFNGR1* were significantly positively correlated with the receptor genes (*FFAR2*, *FFAR3*) of SCFAs, and negatively correlated with the gene (*HDAC9*) that exerts epigenetic regulation in SCFAs, while *BCL2* showed the opposite trend (Figure 8A–C and Figure S1A), which reflects the fact that the metabolism of SCFAs in SAP is influenced by the induction of ICD. We used ssGSEA to further analyze the association of the SCFA-mediated star pathway with hub genes in SAP. Figure 8D,E show that pro-inflammatory immune-related pathways (Th1 cell differentiation, Toll-like receptor signaling pathway, NLRP3 inflammatory vesicles, NF-κB pathway) were positively correlated with *LY96* and *IFNGR1*, while significantly negatively correlated with *BCL2*. Interestingly, *IFNGR1* was significantly positively correlated with almost all inflammatory pathways, the HIF signaling pathway and IL-22BP signaling pathway, fully validating its ability to induce inflammatory outbreaks and the imbalance of multi-organ homeostasis, which is consistent with previous studies and may be related to the production and specific function of IFN-γ (Figure 8F and Figure S1B) [44,45]. The results of molecular docking showed that butyric acid correlated most strongly with hub genes and showed good stability, especially for *BCL2* (binding energy of −3.5 kcal/mol) (Figure 8G–I and Figure S1C–E; Table S1). Overall, all these results suggest that the metabolism and regulatory mechanisms of SCFAs in SAP are closely linked to the drive of ICD.

### 2.7. Clinical and Animal Validation of Hub Genes

**Figure 9:**
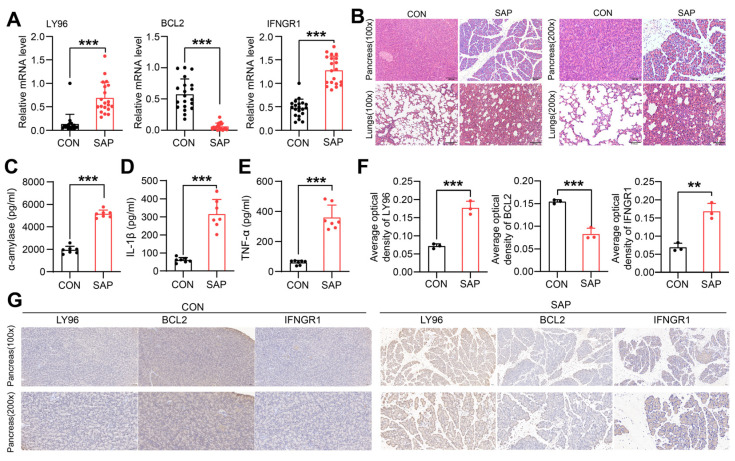
Clinical and animal experimental validation of ICD-related hub genes. (**A**) Relative mRNA expression of the three hub genes in SAP patients versus healthy controls; (**B**) Representative HE-stained micrographs of the pancreas and lungs (original magnification ×100 and ×200); (**C**–**E**) Serum expressional levels of α-amylase, IL-1β, TNF-α by ELISA. (**F**) Immunohistochemical plots showing hub genes in SAP rats and controls (original magnification ×100 and ×200); (**F**) Semiquantitative results of hub genes based on immunohistochemistry. (**G**) Immunohistochemical plots showing hub genes in SAP rats and controls (original magnification ×100 and ×200). Data are shown as mean ± SEM, ** *p* < 0.01, *** *p* < 0.001.

To verify the exact expression levels of ICD-related Hub genes in SAP, we collected and extracted PBMCs for RT-qPCR from 20 clinical SAP patients and healthy controls, respectively. Clinical data showed that patients with SAP present with impaired distal multi-organ function and inflammatory cell infiltration, mainly in the form of acute lung injury (45%), acute liver injury (35%), acute kidney injury (25%), and acute myocardial injury (20%) (Table 1). qRT-PCR analysis showed that *BCL2* expression was significantly downregulated and *LY96* and *IFNGR1* expression was upregulated in SAP patients compared with healthy controls (Figure 9A). Since ICD is initiated by the release of DAMPs by dead alveolar cells in the pancreas, we further explored the expression of hub genes in pancreatic tissue using the SAP rat model. HE staining showed diffuse lobular widening and structural destruction of the pancreas, with extensive inflammatory cell infiltration and hemorrhagic necrosis in the pancreas and lung tissue (Figure 9B). ELISA results demonstrated that serum amylase and inflammatory factor levels were significantly higher in the model group compared to the control group (*p* < 0.001), confirming the successful construction of the model (Figure 9C–E). Interestingly, the results of the IHC were consistent with those of qRT-PCR, which also confirmed that ICD arises in a microenvironment altered by necrotic pancreatic tissue and gradually affects systemic inflammatory signaling. (Figure 9F,G). All these results validate the relevance of the presence of ICD in SAP, which is associated with subsequent tissue destruction and inflammatory storms.

## 3. Discussion

SAP is an immune dysfunctional inflammatory disease in which the uncontrolled inflammatory response accumulates systemically leading to multi-organ failure or infected pancreatic necrosis, which induces serious complications such as acute respiratory distress syndrome, gastrointestinal bleeding and shock [5,46,47]. Despite significant advances in the clinically supportive treatment of SAP in recent years, clarifying the exact pathogenesis of this disease remains a pressing issue. Thus, active research into the microscopic mechanisms of immune imbalance and inflammatory amplification in SAP is essential for improving its prognosis [48]. ICD is a form of programmed cell death that initiates a pattern of immune memory with antigenic and adjuvant properties that induces an excessive immune response in an inflammatory pattern, with sustained immune infiltration and a local “waterfall” inflammatory cascade resulting in a “second strike” on organs and tissues [25,49]. Moreover, ICD has been found to play a key role in the pathogenesis of many non-neoplastic diseases, including autoimmune diseases, myocardial infarction, obesity, and metabolic syndrome, and to influence the development of personalized therapeutic strategies [22,25,50,51].

This study is the first to investigate the evidence for the presence of ICD in SAP and finally identified three pivotal genes (*LY96*, *BCL2*, *IFNGR1*) that play a key regulatory role, which were well validated in samples from both clinical patients and rat models of SAP. *BCL2* expression was downregulated and *LY96* and *IFNGR1* expression was upregulated in SAP patients compared with the healthy controls. *BCL2*, an anti-apoptotic protein, binds to Beclin-1, which improves SAP by inhibiting autophagosome formation [52]. In addition, *BCL2* regulates the permeability of the outer mitochondrial membrane and controls mitochondrial apoptosis. The inhibition of *BCL2* during SAP increases mitochondrial renewal and repair in immature DC cells and macrophages, thus providing sufficient energy support for ICD progression and intercellular communication [53,54]. *LY96*, also known as myeloid differentiation factor 2, acts as an important cofactor in recognizing microbial structural components of lipopolysaccharide by immune cells. It forms a complex with TLR4, a key component of PRRs, to mediate the innate immune response [55,56]. High expression of *LY96* has been proven to be associated with a sustained pro-inflammatory immune response and may be involved in the regulation of disruption of intestinal barrier function and dysbiosis of the microbiota [57]. *IFNGR1*, a key molecule in the interferon signaling pathway, activates macrophages and promotes Th1-mediated inflammatory responses, and the knockdown of *IFNGR1* in mouse models has been associated with recovery from immune dysfunction and an increase in Tregs cells [58,59].

SAP leads to an increased abundance of pathogenic microorganisms and toxic metabolites, which can affect the host pancreatic and intestinal microenvironment [12,15]. Pathogenic microorganisms and their structural components are thought to be a key source of antigenic determinants and exhibit a high degree of antigenicity, forming the basis for initiating ICD in SAP [60,61]. Many cells that die as a result of toxic metabolites from inflammation, toxins, and pathogenic bacteria, emit a series of danger cue signals. The binding of these signaling molecules to PRRs on the surface of host immune cells (with post-translational modified conformations of proteins) leads to an adaptive immune response (executed by cytotoxic T cells) which triggers immune memory [25]. Immune cell infiltration and the production of small molecule metabolites by specific microbiota in the gut have been shown to play a crucial role in the pathogenesis of SAP [62,63].

Therefore, we analyzed the correlation of the identified hub genes with immune infiltration and SCFAs using the ssGSEA algorithm, with a specific focus on the infiltration of immune cells and the activation of pathways associated with adaptive immune response in SAP. The results categorically confirm the necessity of immune factors in the pathogenesis of SAP related to the development and regulation of ICD. Compared with healthy controls, a large infiltration of immune cells was seen in SAP patients, including activated dendritic cells, CD56 bright natural killer cells, gamma delta T (γδT) cells, macrophage and mast cells, suggesting the activation of sustained pro-inflammatory response and the recruitment of inflammatory cells, consistent with previous findings [64,65]. We also found an increased abundance of activated CD4 T cells in the SAP group, which led us to speculate that these types of cells may be related to immune killing during ICD formation. Additionally, the APC-mediated differentiation of T cells appears abnormal, mainly in terms of a discordant ratio of Tregs cells to Th1, Th17, and Th2. Interestingly, the correlation results proved that *LY96* and *IFNGR1* were significantly positively correlated with γδT cells. These are a major histocompatibility complex, unrestricted, innate-like lymphocytes, with unique antigen receptors, that produce cytokines such as IL-17, IFN-γ [66,67]. In addition, γδT cells bridge the innate and adaptive immune systems, which play a central role in host tissue repair, pathogen clearance, inflammatory clearance, and immune surveillance, although they only make up a small proportion of total T cells [68,69,70]. The increased infiltration of γδT cells in patients with SAP may be associated with an inflammatory storm and an increase in the abundance of its causative bacteria. In addition, our group has previously demonstrated the protective role of SCFAs in SAP against multi-organ functional damage [15]. According to the correlation analysis, ICD influenced the regulation of pro-inflammatory immune mechanics and intestinal barrier protection by SCFAs in SAP (particularly against butyric acid) and showed sufficient stability, which still needs to be verified by further basic experiments in the future.

In this study, we integrated a variety of bioinformatics, used several statistical approaches and attempted multiple studies in order to identify the evidence for the presence of ICD in SAP and to uncover associated biomarkers. However, certain limitations still remain. Firstly, this study is retrospective and although we validated the hub genes in clinical patients and rat models, support is still lacking in recent sequencing data, particularly at the single-cell level. Secondly, the exact regulatory mechanisms of ICD in SAP and the correlation between hub genes and the SAP phenotype have not been fully investigated. Finally, due to the limitations of non-oncology database diversity, our analyses relied only on the GEO database and it may be beneficial to include data from other sources in the future.

## 4. Materials and Methods

### 4.1. Data Acquisition and Processing

**Figure 10:**
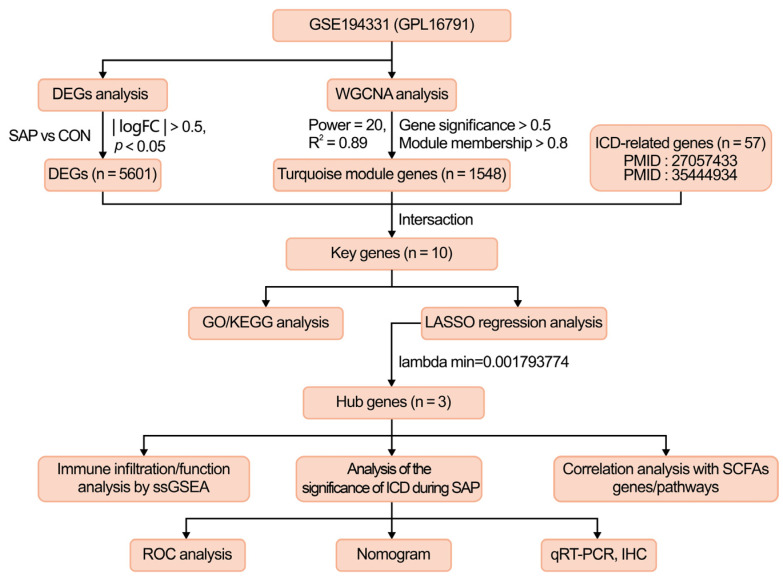
The study flowchart of the whole experiment. GSE, gene expression omnibus series; WGCNA, weighted gene co-expression network analysis; DEGs, differentially expressed genes; ICD, immunogenic cell death; GO, Gene Ontology; KEGG, Kyoto Encyclopedia of Genes and Genomes; LASSO, least absolute shrinkage and selection operator; SCFAs, short-chain fatty acids; ssGSEA, single sample gene set enrichment analysis; IHC, immunohistochemistry.

The methodology for this study is shown in Figure 10. The dataset GSE194331 (platform file GPL16791) was downloaded from the GEO public database (http://www.ncbi.nlm.nih.gov/geo, accessed on 10 March 2022), which contains whole blood gene expression data of 32 healthy patients and peripheral blood gene expression data of 10 SAP patients. After gene re-annotation of the dataset probes through platform files, all data were log-transformed and normalized. The 57 genes related to ICD were sorted according to the published literature, and genes related to SCFAs were extracted from the Genecards database (www.genecards.org/, accessed on 27 March 2022) [26,71]. Please refer to Tables S2 and S3 in Supplementary Materials for details.

### 4.2. Identification and Visualization of Differentially Expressed Genes (DEGs)

DEGs analysis was screened using the Limma package in R [72]. The raw data analyzed were log-transformed after quantile normalization, and genes that met the selected range (|logFC| > 0.5, and adjust *p* < 0.05) were defined as DEGs. The DEGs were then visualized using R packages (pheatmap, dplyr, ggplot2, ggrepel) to plot heatmaps and volcano plots, respectively.

### 4.3. WGCNA

Firstly, the R WGCNA package was used to calculate the correlation coefficients between gene pairs by Pearson’s correlation coefficient to construct the gene co-expression matrix. According to the principle of a scale-free network, soft thresholds (power = 20, R^2^ = 0.89) were selected to construct a scale-free co-expression network in turn, and the adjacency matrix was converted into a topological overlap matrix. Then, cluster analysis was performed to identify gene modules, with a minimum number of 60 genes per module. A dendrogram was constructed using hierarchical clustering to calculate the correlation between the module’s characteristic genes and the disease phenotype, with the module with the highest correlation coefficient and the smallest *p*-value being defined as the disease characteristic. The core genes in the module were filtered by setting the gene significance (GS) > 0.5, and the module membership (MM) > 0.8. (The strongest correlation was for the turquoise module with a maximum correlation value of 0.9 and a minimum *p*-value of 8 × 10^−16^).

### 4.4. LASSO Regression Screening of Central Genes

As a machine learning algorithm, LASSO regression is characterized by its variable selection and complexity regularization while fitting a generalized linear model. The degree of LASSO regression complexity adjustment is controlled by the parameter λ. The larger the value of λ, the greater the penalty for a linear model with more variables, giving a final result that has fewer variables and more representative key genes [43]. LASSO regression analysis of intersecting genes was performed using the glmnet package in R. Then, to determine the best value for λ, we performed ten cross-validations and selected the three most crucial hub genes associated with ICD in this study by the λ selected via the minimum criterion.

### 4.5. Gene Function Enrichment Analysis

R package (clusterProfiler, enrichplot) was used to perform Gene Ontology (GO) (http://geneontology.org/, accessed on 14 April 2022) and Kyoto Encyclopedia of Genes and Genomes (KEGG) (http://www.kegg.jp/ or http://www.genome.jp/kegg/, accessed on 20 April 2022) enrichment analysis of the intersecting genes (DEG, key module genes of the WGCNA screen and ICD-related genes), with *p* < 0.05 considered to be a significantly different level for the enrichment result [73,74].

### 4.6. ssGSEA

The ssGSEA enrichment analysis was performed using the R GSVA package. Firstly, the percentage of immune cell infiltration and the activity score of immune/SCFA-related pathways were calculated for each sample based on the expression matrix of the patient samples in the SAP group. The differences in the proportion of immune cell infiltration and the activity of immune/SCFA-related pathways between the SAP group and healthy controls were compared using the vioplot package in R. Secondly, the R (ggplot2 and reshape2) packages based on Spearman’s rank correlation were used to compare the expression levels of hub genes in the SAP group with the proportion of immune cell infiltration and immune/SCFA-related pathways. The final visualization is presented using heatmaps, box plots, and lollipop charts.

### 4.7. Differential Expression Analysis and Receiver Operating Characteristic (ROC) Curve Validation

R (limma and ggpubr) packages were used to analyze and compare the expression levels of the SAP group with those of the control group regarding the final hub genes and displayed via boxplots. Meanwhile, ROC curve analysis was performed for each hub gene using the R pROC package, and the area under the curve (AUC) with a 95% confidence interval (CI) was calculated. The significance of ICD was judged by the AUC value, with values close to 1 indicating higher accuracy of the model training.

### 4.8. Nomogram Construction and Verification

A nomogram was constructed using the R software with the “rms” package to illustrate the interactions between the three Hub genes most associated with ICD occurrence in SAP [75]. “Points” indicate the scores of a candidate gene, and “Total Points” represents the sum of the above scores for all genes. The calibration curve and C-index were used to assess the performance of the nomogram and to visually reflect how well the predicted probabilities matched the observed probabilities, thus demonstrating the vital value of the ICD in SAP.

### 4.9. Peripheral Blood Mononuclear Cells (PBMCs) Collection and qRT-PCR

The research protocols for this study were approved by the Ethics Committee of the First Affiliated Hospital of Dalian Medical University (ethics number: PJ-KY-2021-42) and included a total of 20 patients with SAP and healthy controls, respectively. All included SAP patients met the 2012 revised Atlanta criteria and all included study populations gave informed consent including the details of this study (Tables S4–S6) [76]. SAP is an inflammatory disease that has spread to multiple organs throughout the body, and monocytes are an essential component of the body’s immune defense system. Therefore, to ensure consistency with the sample source of the sequencing data, PBMCs were extracted from the peripheral blood of SAP patients to detect the expression of key genes. Briefly, fasting 5 mL peripheral blood samples were collected from patients and healthy controls using anticoagulant tubes and PBMCs were separated by density gradient centrifugation within one hour using Ficoll-Paque Plus (Cytiva, GE Life, Louisville, KY, USA) according to the manufacturer’s instructions. RNA was extracted from PBMCs based on the Trizol method. Servicebio RT First Strand cDNA Synthesis Kit (Servicebio, G3330, Wuhan, China) was used to perform reverse transcription, and PCR system kit 2×SYBR Green qPCR Master Mix (None ROX) (Servicebio, G3320, Wuhan, China) was used in RT-PCR reaction. The 2^−ΔΔCt^ method was used to analyze the relative expression of the hub genes in the two groups and set at 1.0 in the healthy control group. The sequence of the primers was as follows (Table 2).

### 4.10. Establishment of Rat SAP Model

Specific pathogen-free (SPF) grade male SD rats (6–8 weeks old, approximately 220 g) were purchased from the Laboratory Animal Centre of Dalian Medical University (License No, SCXK (Liao) 2018-0003). All rats were housed in a specific SPF environment (temperature: 22 ± 2 °C, humidity: 45 ± 5%, 12 h light/dark cycle) and were provided with unrestricted food and water. All animal care and experimental procedures were approved by the Research and Animal Ethics Committee of Dalian Medical University (Dalian, China) (AEE19003). We also followed the Guidelines for the Care and Use of Laboratory Animals developed by the National Institutes of Health, USA and all animals were adaptively fed for 1 week before molding.

Rats were randomly divided into a control group (CON) and a SAP group, with seven rats in each group. The rats in the SAP group were constructed according to previous studies, with retrograde injection of 5% sodium taurocholate into the pancreaticobiliary duct to create the SAP model [77]. Twenty-four hours after treatment, arterial blood samples were collected from the abdominal aorta, centrifuged at 4 °C, 3000 rpm, for 10 min and the serum was collected and stored at −80 °C. Rat pancreas and lung tissues were collected, fixed in 10% formalin and embedded in paraffin for subsequent staining experiments.

### 4.11. Histology and Immunohistochemistry

The fixed pancreatic and lung tissues were paraffin-embedded, stained with hematoxylin and eosin (H&E) solution, then observed and photographed under a light microscope (Olympus, Japan). According to the previous study, the pancreas was scored based on the severity of the histological lesion on a scale of 0 to 16 [78]. IHC staining was performed according to standard embedding, sectioning, dewaxing, and rehydration procedures. Briefly, sections were incubated overnight at 4 °C with primary antibody (anti-*BCL2*, 1:200, Abclonal, A11313; anti-*LY96*, 1:500, Affinity, #DF6669; anti-*IFNGR1*, 1:200, Abclonal, A5748) and incubated with biotinylated goat anti-rabbit IgG secondary antibody at 37 °C for 1 h. The sections were then incubated with peroxidase for 15 min at 37 °C, followed by staining with 3,3′-diaminobenzidine. Hematoxylin (Solarbio Science & Technology, Beijing, China) was used to counterstain the sections. Images were observed and collected under a light microscope (Olympus, Japan). Finally, the sections were analyzed semi-quantitatively using a quantitative digital image analysis system (image-pro Plus 6.0; Media Cybernetics, Inc., Rockville, MD, USA).

### 4.12. Serum Amylase and Inflammatory Factor Assay

Amylase levels in serum were measured using an alpha-amylase assay kit (Jiancheng Bioengineering Institute, Nanjing, China) according to the manufacturer’s instructions. The levels of IL-1β and TNF-α in serum were determined by commercial enzyme-linked immunosorbent assay (ELISA) kits (Westang Bio-tech Co., Ltd., Shanghai, China). The operation was performed following the ELISA kit instructions.

### 4.13. Statistical Analysis

Data results are expressed as the mean ± standard error of the mean (SEM), and in vivo validation of clinical and animal specimens of the hub genes included at least three independent experiments with replicated samples. Statistical analysis of data from this study was carried out using R language (Version 4.1.1; R Core Team, 2021). The Student’s *t*-test was used to compare two groups of normally distributed data, whereas the Wilcox Rank Sum test was used for the comparison of non-normally distributed data and was expressed as the median (interquartile range). The chi-square test was used for categorical variable data between the two groups, and the Spearman rank correlation test was applied to analyze the correlation of hub genes with immune infiltration, immune pathways, and metabolic genes/pathways of SCFAs. The level of statistical significance was set at *p* < 0.05.

## 5. Conclusions

In conclusion, we introduce the novel concept of sustained pro-inflammatory immunity, triggered by ICD, having a role in SAP. Based on a comprehensive bioinformatics analysis, we have mined and validated three pivotal ICD-related genes (*LY96*, *BCL2*, *IFNGR1*) that play a critical role in SAP. Importantly, our study also analyzed the impact of ICD formation on immune infiltration and metabolism of SCFAs using the ssGSEA algorithm, which is essential for studying the mechanisms of immune imbalance in SAP complicated by multi-organ failure and is expected to be a new direction for future exploration of immunomodulatory intervention during SAP.

## Figures and Tables

**Table 1:** Clinical characteristics of the SAP and CON groups.

Characteristic	CON (n = 20)	SAP (n = 20)	*p*-Value
Gender (F/M)	10/10	7/13	0.337 * ^N.S^
Age (years)	62.60 ± 14.652	57.55 ± 11.989	0.240 * ^N.S^
PaO_2_ (mmHg)	78.09 ± 5.128	71.13 ± 10.732	0.014 **
FiO_2_ (%)	21.00 ± 0.000	27.60 ± 7.816	0.001 ***
Oxygenation index (mmHg)	371.86 ± 24.420	285.114 ± 107.183	0.002 ***
SpO_2_ (%)	94.10 ± 1.889	83.45 ± 7.708	0.000 ***
WBC (10^9^/L)	5.488 ± 1.147	14.443 ± 5.120	0.000 ***
PCT (ng/mL)	0.12 ± 0.110	1.95 ± 3.088	0.016 **
CK-MB (ug/L)	1.27 ± 1.015	2.93 ± 2.723	0.018 **
Hs-Tnl (ng/mL)	0.031 ± 0.047	0.26 ± 0.594	0.102 * ^N.S^
ALT (U/L)	18.55 ± 10.516	159.50 ± 217.789	0.009 ***
AST (U/L)	24.10 ± 5.261	186.05 ± 323.926	0.038 **
TBIL (umol/L)	15.89 ± 3.817	52.35 ± 65.475	0.022 **
DBIL (umol/L)	1.72 ± 1.293	26.50 ± 44.053	0.021 **
γ-GGT (U/L)	22.60 ± 12.672	188.90 ± 315.916	0.030 **
Creatinine (umol/L)	61.20 ± 17.243	103.20 ± 79.348	0.031 **
Urea (mmol/L)	5.53 ± 1.063	9.97 ± 13.521	0.159 * ^N.S^
Amylase (U/L)	63.80 ± 17.255	1135.00 ± 1581.329	0.007 ***
Lipase (U/L)	98.55 ± 55.457	2018.58 ± 1690.193	0.000 ***
Serum calcium (mmol/L)	2.16 ± 0.113	1.79 ± 0.413	0.001 ***

^N.S^ not significant, * *p* < 0.1, ** *p* < 0.05, *** *p* < 0.001. SAP, severe acute pancreatitis; CON, healthy controls; WBC, white blood cell; PCT, procalcitonin; CK-MB, creatinine kinase-myocardial band; Hs-Tnl, high-sensitivity troponin I; ALT, alanine aminotransferase; AST, aspartate aminotransferase; TBIL, total bilirubin; DBIL, direct bilirubin; γ-GGT, gamma-glutamyltransferase.

**Table 2:** The Sequences of Primers.

Genes	Forward Primer (5′-3′)	Reverse Primer (5′-3′)
GAPDH	CCTCGTCCCGTAGACAAAATG	TGAGGTCAATGAAGGGGTCGT
BCL2	GGAAGCTTGTCATCAATGGAAATC	TGATGACCCTTTTGGCTCCC
LY96	CATGAATCTTCCAAAGCGCAA	CTCCCAGAAATAGCTTCAACAACA
IFNGR1	CAGAAAGGAGGAGAAGCAAATCA	ATCTCACTTCCGTTCATTCTCACAT

## Data Availability

The datasets presented in this study can be found in online repositories. The names of the repository/repositories and accession number(s) can be found in the article/Supplementary Material.

## Supplementary Materials

The supporting information can be downloaded at: https://www.mdpi.com/article/10.3390/ijms24033033/s1.
