# Supplementary material for: Identification of Key Biomarkers Associated with Immunogenic Cell Death and Their Regulatory Mechanisms in Severe Acute Pancreatitis Based on WGCNA and Machine Learning
Source: Int J Mol Sci. 2023 Feb 3;24(3):3033. doi: 10.3390/ijms24033033 (PMC9918120; doi:10.3390/ijms24033033)
Supplement: Supplementary file 1 [file ijms-24-03033-s001.zip › ijms-2154062-supplementary.pdf]

Supplementary Materials

Supplementary methods

Molecular docking and kinetic simulations

Download the 3D structures of SCFAs in sdf format from the PubChem database (https://pubchem.ncbi.nlm.nih.gov) according to their CAS numbers (acetic acid, 64-19-7; Propionic acid, 79-09-4; butyric acid, 107-92-6), import them into ChemBio3D Ultra 14.0 for energy minimisation, set the Minimum RMS Gradient to: 0.001 and save the small molecules in mol2 format. The optimised SCFAs are imported into AutodockTools-1.5.6 for hydrogenation, charge calculation, charge assignment, setting of rotatable keys and then saved in "pdbqt" format. The protein structures of LY96 (PDB ID: 2E56), BCL2 (PDB ID: 5FCG) and IFNGR1 (PDB ID: 1FYH) were downloaded from the PDB database (http://www.rcsb.org/); Pymol 2.3.0 was used to remove protein crystalline water, raw ligands, etc. The protein structures were imported into AutoDocktools (v1.5.6) for hydrogenation, charge calculation The protein structures were imported into AutoDocktools (v1.5.6) for hydrogenation, charge calculation, charge assignment, atom type assignment and saved in "pdbqt" format. AutoDock Vina1.1.2 was used to perform the docking and PyMOL2.3.0 was used to analyse the interaction patterns of the docking results.

Molecular dynamics simulations were performed using Gromacs 2020.1, in which charm36-mar2019 force field was chosen. The protein (LY96, BCL2, IFNGR1) and molecule complex (butyric acid) were solved with TIP3P water and immersed in a dodecahedron box extending to at least 1 nm of the solvent on all sides. Also, the system was neutralized by Na+ and Cl-, then added 0.15 M NaCl. It was energy minimized by using the steepest descent algorithm for 5000 steps, and it made a maximum force of less than 1000 kJ/mol/nm. After energy minimization, the system was equilibrated in a constrained NVT (number of particles, volume, temperature) and NPT (number of particles, pressure, temperature) running for 100 ps. NVT equilibration ensured the system was brought to the desired temperature (300K), with which we seek to establish the proper orientation about the protein. After NVT equilibration, we stabilize the pressure of the system under an NPT ensemble. Through NVT and NPT equilibration, it was well-equilibrated at 300 K and 1 bar. Finally, MD simulations of the complex were carried out for 100 ns. Trajectories were saved every 10 ps for analysis. The Verlet cut-off scheme and a Leap-frog integrator with a step size of 2 fs were applied. For temperature coupling, the modified Berendsen thermostat and the Parrinello-Rahman barostat for pressure coupling were used. For long-range electrostatic interaction, the Particle Mesh Ewald method was used.

The root-mean-square displacement (RMSD) was calculated by GROMACS 2020.1. The binding free energies of the complex between the protein and molecule were calculated using gmx_mmpbsa tool (https://github.com/Jerkwin/gmxtool/tree/master/gmx_mmpbsa) based on the molecular mechanics/Poisson-Boltzmann surface area (MM/PBSA) method.

Supplementary tables

Table S1. MM-PBSA calculation of the average binding energy between Bcl2 and butyric acid and its components.

Active force	Combined energy (kcal/mol)
ΔEvdw	-42.16 ± 4.98
ΔEelec	-6.57 ± 1.47
ΔGpolar	35.11 ± 4.04
ΔGnonpolar	-9.52 ± 0.63
ΔGtotal	-23.14 ± 2.27

The total free energy of binding of *BCL2* to butyric acid was -42.16 kcal/mol, with van der Waals forces accounting for the most, followed by non-polar interactions and electrostatic interactions, with polar interactions being detrimental to the binding of both. The smaller the ΔGtotal, the greater the likelihood of union.

Table S2. List of 57 ICD-related genes.

ENTPD1	PDIA3	IFNA1	TLR4	IL1A	TLR3	AIM2
NT5E	EIF2AK3	CASP1	FOXP3	IL33	TLR7	AGER
CALR	PIK3CA	IL1R1	IFNG	ROCK1	TLR9	TREM1
HMGB1	FNB1	IL1B	IFNGR1	PANX1	CLEC4E	FPR1
HSP90AA1	CXCR3	NLRP3	IL17A	BCL2	CLEC7A	FPR2
ATG5	IL10	P2RX7	IL17RA	PPIA	DDX58	P2Y2R
BAX	IL6	LY96	PRF1	HSPA4	IFIH1	P2Y6R
CASP8	TNF	MYD88	HMGN1	TLR2	CGAS	P2Y12R
CASR						

Table S3. List of SCFAs-related genes.

CYP7A1	FFAR2	FFAR3	HDAC1	HDAC9	PYY	ACSS2

Table S4. Definition of diagnosis of acute pancreatitis.

- Abdominal pain consistent with acute pancreatitis (acute onset of a persistent, severe, epigastric pain often radiating to the back);
- Serum lipase activity (or amylase activity) at least three times greater than the upper limit of normal;
- Characteristic findings of acute pancreatitis on contrast-enhanced computed tomography (CECT) and less commonly magnetic resonance imaging (MRI) or transabdominal ultrasonography.

Table S5. Grading the severity of acute pancreatitis.

- Mild acute pancreatitis
  - No organ failure
  - No local or systemic complications
- Moderately severe acute pancreatitis
  - Organ failure that resolves within 48 h (transient organ failure) and/or
  - Local or systemic complications without persistent organ failure

- Severe acute pancreatitis<br>- Persistent organ failure (>48 h)<br>- –Single organ failure<br>- –Multiple organ failure

Table S6. Modified Marshall scoring system for organ dysfunction.

Organ system	Score				
	0	1	2	3	4
Respiratory (PaO_2/FiO_2)	>400	301–400	201–300	101–200	≤101
Renal*					
(serum creatinine, μmol/l)	≤134	134–169	170–310	311–439	>439
(serum creatinine, mg/dl)	<1.4	1.4–1.8	1.9–3.6	3.6–4.9	>4.9
Cardiovascular (systolic	>90	<90, fluid	<90,	<90,	<90,
For non-ventilated patients, the FiO_2 can be estimated from below:					
**Supplemental oxygen**	**FiO_2 (%)**				
Room air	21				
2	25				
4	30				
6-8	40				
9-10	50				

- A score of 2 or more in any system defines the presence of organ failure.
- * A score for patients with pre-existing chronic renal failure depends on the extent of further deterioration of baseline renal function. No formal correction exists for a baseline serum creatinine ≥134 μmol/l or ≥1.4 mg/dl.
- † Off inotropic support.

Supplementary figures

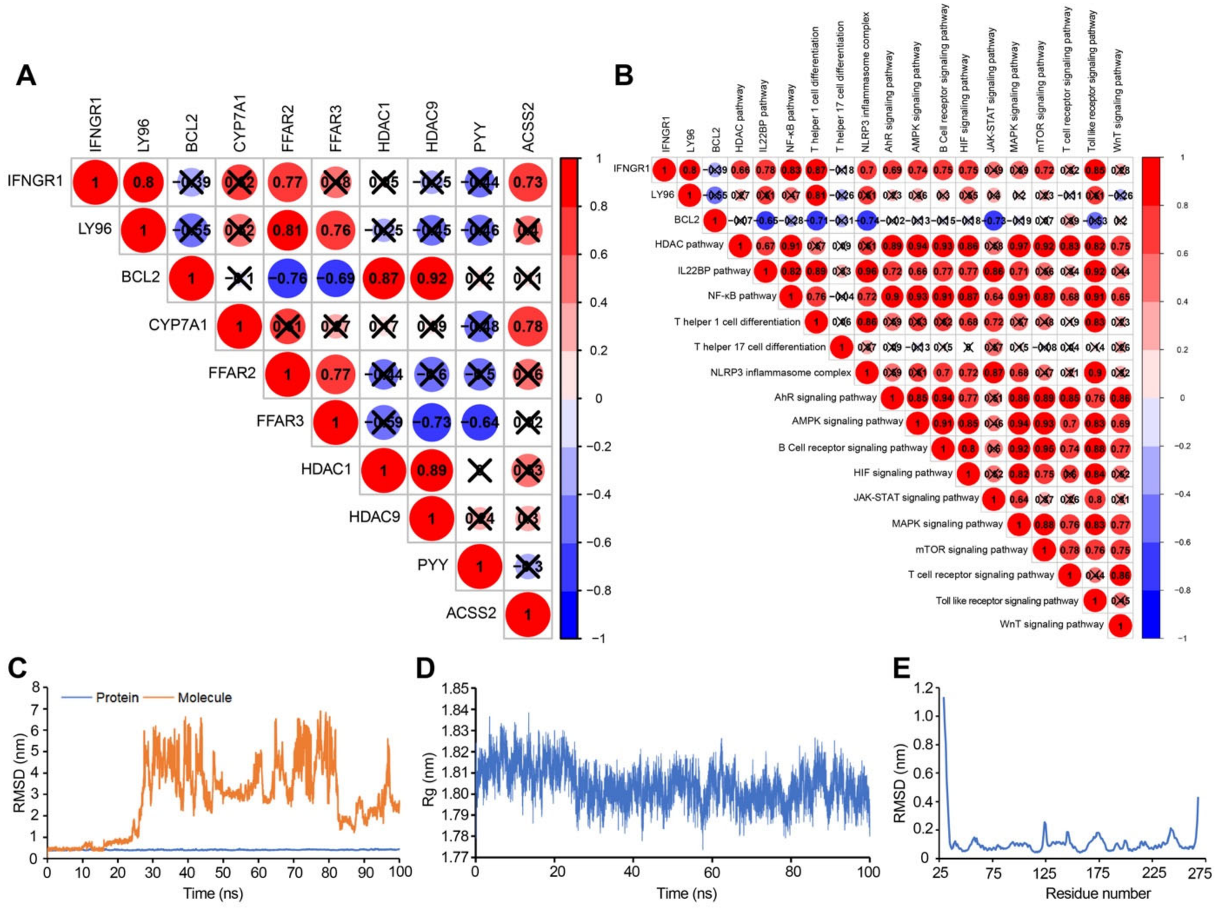

Figure S1. Correlation analysis of Hub genes with SCFAs. (**A**,**B**) Heatmaps showing hub genes and genes/pathway related to the metabolism of SCFAs. The numbers represent correlation coefficients and the crosses indicate no correlation. (**C**) Root mean square deviation (RMSD) of butyric acid and *BCL2* during the simulated 100 ns. As shown, the RMSD of the *BCL2* structure was very stable during the simulation and the RMSD of butyric acid reached equilibrium after 30 ns. (**D**) Gyration radius (Rg) of the *BCL2* skeleton atom for the simulated 100 ns process. (**E**) Simulated root mean square fluctuations (RMSF) of *BCL2* alpha-C over the course of 20–100 ns. The radius of gyration was very stable during the simulation. The α-C atoms of the amino acids fluctuated less during the simulation, except for the N-terminal amino acids, which fluctuated more, and the combined figures **C**–**E** show that the *BCL2* was structurally very stable during the simulation.
